# Development of Plant–Fungal Endophyte Associations to Suppress Phoma Stem Canker in *Brassica*

**DOI:** 10.3390/microorganisms9112387

**Published:** 2021-11-19

**Authors:** Davood Roodi, James P. Millner, Craig R. McGill, Richard D. Johnson, Shen-Yan Hea, Jenny J. Brookes, Travis R. Glare, Stuart D. Card

**Affiliations:** 1 Resilient Agriculture, AgResearch Limited, Grasslands Research Centre, Private Bag 11008, Palmerston North 4410, New Zealand; roodi_dave@yahoo.com (D.R.); Richard.Johnson@agresearch.co.nz (R.D.J.); 2 School of Agriculture & Environment, Massey University, Private Bag 11222, Palmerston North 4442, New Zealand; J.P.Millner@massey.ac.nz (J.P.M.); C.R.McGill@massey.ac.nz (C.R.M.); 3 Khorasan Razavi Agricultural and Natural Resources Research and Education Center, Agricultural Research, Education and Extension Organization (AREEO), Mashhad 91769-83641, Iran; 4 Digital Agriculture, AgResearch Limited, Invermay Agricultural Centre, Private Bag 50034, Mosgiel 9053, New Zealand; Shen.Hea@agresearch.co.nz; 5 Bio-Protection Research Centre, P.O. Box 85084, Lincoln University, Lincoln 7647, New Zealand; Jenny.Brookes@lincoln.ac.nz (J.J.B.); Travis.Glare@lincoln.ac.nz (T.R.G.)

**Keywords:** *Beauveria bassiana*, biological control, brassicaceae, cordycipitaceae, leptosphaeriaceae, *Leptosphaeria maculans*, phytopathogen

## Abstract

Endophytic microorganisms are found within the tissues of many plants species, with some conferring several benefits to the host plant including resistance to plant diseases. In this study, two putative endophytic fungi that were previously isolated from wild seeds of *Brassica*, identified as *Beauveria bassiana* and *Pseudogymnoascus pannorum*, were inoculated into cultivars of three *Brassica* species—*Brassica napus*, *Br. rapa* and *Br. oleracea*. Both fungal endophytes were reisolated from above- and below-ground tissues of inoculated plants at four different plant-growth stages, including cotyledon, one-leaf, two-leaf, and four-leaf stages. None of the plants colonised by these fungi exhibited any obvious disease symptoms, indicating the formation of novel mutualistic associations. These novel plant–endophyte associations formed between *Brassica* plants and *Be. bassiana* significantly inhibited phoma stem canker, a devastating disease of *Brassica* crops worldwide, caused by the fungal pathogen *Leptosphaeria maculans*. The novel association formed with *P. pannorum* significantly suppressed the amount of disease caused by *L. maculans* in one out of two experiments. Although biological control is not a new strategy, endophytic fungi with both antiinsect and antifungal activity are a highly conceivable, sustainable option to manage pests and diseases of economically important crops.

## 1. Introduction

Crops from the genus *Brassica* were among the earliest plants to be widely cultivated by mankind [1]. *Brassica* displays enormous diversity and subsequently provides the widest assortment of products used by man from a single plant genus [2] with many parts of the plant being edible, including their buds, flowers, leaves, roots, seeds, stems and tubers [3,4]. Many species of *Brassica* are used as important animal and human food sources, as ornamentals, sources of medicines, soil conditioners, green manures, composting crops, and are valued in bioremediation and in the production of edible and industrial oils [2,3,5,6]. The cultivation of oilseed rape (*Brassica napus*) has now become the second most important oilseed crop after soybean, with a worldwide production of over 70 million metric tonnes in 2018 [7] with Canada, China and India amongst the top producers.

All the economically important species of *Brassica*, including oil or leafy types, are vulnerable to attack from a wide range of invertebrate pests and plant pathogens [8,9,10]. One of the most important fungal pathogens of oilseed rape is *Leptosphaeria maculans*, which causes phoma stem canker (also known as blackleg disease) [11,12]. This ascomycete causes large yield losses and is a major constraint in the production of oilseed rape in Australia, Europe, New Zealand and North America. In the UK alone, the disease can cause up to GBP 56 million worth of damage per season [13] and more than USD 900 million per season worldwide [14]. Integrated disease management practices, including crop rotation and stubble management, are recommended to control the pathogen, with many growers also routinely relying on chemical fungicides to minimise crop losses [11,12,15]. Although the use of resistant canola varieties was seen as promising, field populations of *L. maculans* can overcome major resistance genes within a few years, potentially accelerated by growers that have adopted shorter crop rotations to capitalise on the high profits from the crop [15].

Overuse of agrochemicals has extensively damaged our environment and contributed to steep losses in biodiversity [16,17]. In direct response, the European Union has now banned three types of neonicotinoids, systemic pesticides commonly used as a seed coat on many crops, due to their negative impact on pollinators vital for food production, e.g., bees [18]. Coupled with chemical resistance problems [19], and negative impacts on human health [17], this advocates the need for new and innovative pest and disease management strategies for crops including *Brassica*.

Symbiotic microorganisms that live inside plant tissues, termed endophytes, associate with the majority of plant species grown in natural and managed ecosystems [20,21]. Species of Brassicaceae are no exception, with many of their associated symbionts classified as mutualistic, conferring one or several advantageous traits to their hosts including improved plant growth, increased yield and resistance against pests and/or plant pathogens (reviewed by [22]). Biological control of plant pathogens is not a new concept [23], but the transfer of beneficial microbial endophytes from wild plant species to modern day cultivars to obtain additional traits is a novel strategy that may overcome many of the dilemmas faced by traditional biological control approaches [20]. This study focused on the colonisation of cultivated *Brassica* species by two fungal endophytes of wild *Brassica* and investigated whether this novel association could offer plant resistance against the disease of phoma stem canker.

## 2. Materials and Methods

### 2.1. Fungal Endophytes Isolated from Wild Brassica

Two accessions of *Brassica rapa* were imported on permit no. 2015058982 as seed into the Margot Forde Germplasm Centre, New Zealand’s national genebank of grassland plants. The accessions were catalogued as O2380 and O2377 and stored at 0 °C and 30% relative humidity until use. Both accessions were collected as wild material and originated from California, USA. Seeds from these accessions were subsequently screened for the presence of microbial endophytes according to a modified method of Roodi et al. [24]. Briefly, seed were surface disinfected and grown under sterile conditions within tissue culture pots containing Murashige & Skoog (MS) basal salts [25] with minimal organics (Sigma-Aldrich, Auckland, New Zealand), plus 3% sucrose and 1.5% agar [26]. After incubation, clean seedlings (i.e., those free of epiphytic microbial growth when inspected with a dissecting microscope (Carl Zeiss AG, Oberkochen, Germany)) were dissected into two components: shoot and root. These organs were further dissected into 2–3 mm^2^ pieces using sterile forceps and a scalpel. Ten pieces per organ type from each seedling were transferred to Petri plates containing PDA. Petri plates were incubated for 3 weeks at 22 °C in the dark and checked daily under a dissecting microscope for microbial growth. Fungal colonies arising from dissected tissue pieces were selected, subcultured and purified. Representative fungal isolates were then subcultured onto fresh PDA and stored on sterilized wheat grain submersed in 20% glycerol within an ultralow temperature freezer (ULT) at −80 °C (according to [27]).

Species identification of fungal endophytes was achieved initially by morphological examination of reproductive structures on water agar using a BX50 microscope and DP12 digital camera system (Olympus NZ Ltd., Auckland, New Zealand). Confirmation of species identity was conducted by PCR amplification of the Internal Transcribed Spacer (ITS) of rDNA gene sequences [28]. For PCR, both fungal isolates were incubated at 22 °C for two weeks and their DNA extracted using Quick-DNA^TM^ Fungal/Bacterial Kit (Zymo Research Corporation, Irvine, USA) and quantified using the Invitrogen Qubit^TM^ 4 Fluorometer (ThermoFisher Scientific Inc., Waltham, MA, USA). The PCR reaction contained 1 μL of DNA extract suspension (15–20 ng/μL) of each purified fungal colony, 5 μL 10X PCR buffer, 1.5 μL MgCl_2_ (50 mM), forward primer, ITS1 (5′ TCCGTAGGTGAACCTGCGG-3′) and reverse primer ITS4 (5′-TCCTCCGCTTATTGATATGC-3′), 0.4 μL dNTPS (25 mM), 0.25 μL Taq-polymerase and 39.85 μL sterile Milli-Q water, to make a 50 μL PCR reaction. PCR was performed in a thermocycler (Bio-Rad C1000 Touch^TM^, Bio-Rad Laboratories Inc., Hercules, USA) with the following conditions: an initial step of 95 °C for 5 min was followed by 36 cycles of 94 °C for 30 s, 56 °C for 30 s, 72 °C for 90 s and a final step of 72 °C for 10 min. The reaction mixture from each sample was electrophoresed on a 1.5% agarose gel containing ethidium bromide. The gel was viewed on a transilluminator (Gel Doc™ XR+, Bio-Rad Laboratories Inc., Hercules, USA) to identify samples with amplification products. These products were purified and concentrated using the DNA clean & concentrator kit (Zymo Research Corporation, Irvine, USA) prior to Sanger sequencing [29] (New Zealand Genomics Ltd., Dunedin, New Zealand). DNA sequences were analysed with the software package Geneious Prime^®^ version 2019.1.1 (Biomatters Ltd., Auckland, New Zealand). Sequences greater than 600 bp were used in BLASTn searches against the NCBI nonredundant database, and those with greater than 98% identity were selected and named.

Further identification of *Beauveria* sp. was achieved via the 1-alpha (EF1-apha) elongation factor using primers EF-1 (forward) 5′ ATGGGTAAGGAGGACAAGAC and EF-2 (reverse) 5′ GGAAGTACCAGTGATCATGTT [30] with a 25 μL PCR reaction mixture that consisted of: 15.75 μL sterile water, 2.5 μL buffer (10×) plus MgCl_2_ (2 mM), 2 μL deoxynucleotide (dNTP’s) (2.5 mM), 0.25 μL Fast start polymerase Taq, 1 μL of each primer, 0.5 μL bovine serum albumin (Bio Labs^®^ Inc., Lawrenceville, USA) and 2 μL of extracted DNA per sample. Thermocycling conditions were set as 95 °C for 5 min followed by 40 cycles of (95 °C for 45 s, 53 °C for 45 s, 72 °C for 1 min) and a final extension of 72 °C for 7 min. Elongation factor sequences were aligned with the programme ClustalW in the software package Geneious Prime^®^, with a gap cost set at 15. The sequence of strain O2380 was compared to previously published sequences, including those referred to by Rehner and Buckley [31], downloaded from GenBank. Sequences were aligned and trimmed to 687–707 bp for each isolate. After alignment, a tree was generated using Geneious Tree Builder, set to the Jukes-Cantor distance model and Neighbour-Joining with *Be. hoplocheli* Bt124 designated as an outgroup. Bootstrapping was set at 1000 replicates.

### 2.2. Development of Novel Brassica–Endophyte Associations

Seeds from three forage cultivars of *Brassica*, cv. Hunter, Titan and Regal, were sourced from PGG Wrightson Seeds Ltd. (Christchurch, New Zealand) for the development of novel, or artificial, plant–endophyte associations. Hunter, a leafy turnip, is an interspecific hybrid developed by crossing turnips with related Asiatic leaf vegetables of the same species. Titan, a forage rape, is an interspecies cross developed by crossing rape with kale, while Regal is a high-yielding, intermediate-height kale. In order to reduce any epiphytic microorganisms attached to the seed coats, seeds were surface disinfected by washing for five min in 5% aqueous Tween^®^ 20 solution (Sigma-Aldrich Inc., Auckland, New Zealand), two min in 70% ethanol, 10 min in 2% sodium hypochlorite, one min in 70% ethanol and were rinsed three times in sterile tap water. Seeds were then dried on filter paper (110 mm, ThermoFisher Scientific Inc., LabServ^®^, Waltham, MA, USA) within a sterile environment and stored at 4 °C until use.

The two candidate fungi were removed from the ULT freezer and defrosted at room temperature before plating onto PDA (CM0139, Oxoid Ltd., Basingstoke, UK). Petri plates containing the fungi were then incubated for approximately two weeks at 22 °C in the dark to promote mycelial growth and sporulation. Subsequent spores of each fungal strain were dislodged by adding 50 mL of sterile water to the Petri plate and gently brushing the fungal colony with a sterile loop. The resulting crude suspension was passed through a single layer of sterile Miracloth (Sigma-Aldrich Inc., Auckland, New Zealand) to remove mycelial fragments, and one drop of Tween-20^®^ was added to the solution to stop the spores adhering to each other. The concentration of each spore suspension was estimated using a haemocytometer and adjusted to 10^6^ spores per mL. The viability of fungal spores was assessed by spraying diluted aliquots of the prepared spore suspensions onto fresh PDA and counting the subsequent colonies after 3 days of incubation at 22 °C in the dark.

### 2.3. Inoculation of Brassica Seed with Fungal Spore Suspensions

Disinfected seeds of each *Brassica* cultivar were soaked in a spore suspension of each fungal endophyte strain for 10 min at room temperature. Control seeds were soaked in aqueous Tween-20^®^ solution. All treated and control seed then were transferred to sterile filter papers and allowed to dry at room temperature for 30 min. Seeds were sown in a vermiculite growth medium supplemented with essential nutrients to support plant growth, including necessary macro- and micro-nutrients in the form of a nutrient solution, following the manufacturer’s instructions (Thrive^®^, Yates New Zealand Ltd., Auckland, New Zealand). In each pot, measuring 10 × 15 cm, 10 seeds were sown, which were later thinned to three seedlings per pot after germination. Pots were subsequently placed in a glasshouse with natural light at 20–25 °C, and watered as required. Plant health, development, and any visible disease symptoms were assessed daily under a stereomicroscope.

### 2.4. Assessment of Endophyte Colonisation

The colonisation frequencies of the forage *Brassica* cultivars inoculated by either of the two fungal endophytes was determined in root and shoot (stem) tissues at four different plant growth stages, (1) cotyledon, (2) one-leaf, (3) three-leaf and (4) the four-leaf growth stage. For each of the two endophytes, for each *Brassica* cultivar at each growth stage, eight inoculated and eight uninoculated (control) plants were assessed as follows; each plant was removed from the growth medium, washed in tap water, and surface disinfected by washing in 5% aqueous Tween-20^®^ solution for five min, 70% ethanol for 2 min, 5 min in 1% sodium hypochlorite followed by 70% ethanol for one min and finally three rinses in sterile tap water. At the cotyledon and one-leaf stage, a modified surface disinfection protocol was utilised as the above protocol was proven to be too harsh on the delicate plant tissues. In this protocol, 70% ethanol was used for one min and 30 s rather than two minutes and one minute. To assess the efficacy of the surface disinfection protocols, 3 × 20 μL drops of tap water from the last rinse were plated onto PDA and incubated at 22 °C. These PDA plates were subsequently observed every day for two weeks under a dissecting microscope for microbial growth. Following surface disinfection, seedlings were dried on filter papers (110 mm, LabServ^®^, Waltham, USA) within a sterile environment for 30 min at room temperature. Plant tissues were dissected from each plant with a sterile scalpel, and ten 1–2 mm^2^ pieces (of each root and shoot) transferred to Petri plates containing PDA. Petri plates were incubated at 22 °C and checked regularly (for up to three weeks) with the aid of a dissecting microscope for microbial growth. The presence of each fungal endophyte was assessed visually using a dissecting microscope. The number of fungal colonies that emerged from each tissue piece was recorded and the data presented as a percentage of tissue colonisation frequency (TCF) for each endophyte.

Confirmation of endophyte species was assessed using PCR as previously described. Statistical analyses for the endophyte frequency data were performed using the software R [32]. A generalised linear model (GLM) with a binomial distribution and logit link function was used to model the proportion of endophyte-infected tissue pieces (number of infected tissue pieces/total number of tissue pieces) for each fungal endophyte strain. The fixed effects used in the model were the growth stage, shoot/root location and *Brassica* cultivar. All two-way interaction terms between the variables were included in the model. Analysis of Deviance was used to assess the significance of the fixed effects and their interaction terms. The R package “emmeans” was used to generate back-transformed probabilities, standard errors and 95% confidence limits [33].

### 2.5. Bioactivity of Fungal Endophytes towards Leptosphaeria Maculans

The bioactivity of the novel *Brassica*–endophyte associations was assessed against *L. maculans* (strain LUPP2376), a highly pathogenic strain originally identified from a diseased swede collected in Gore, New Zealand [34], supplied by Dr Eirian Jones, Lincoln University. After subculturing on PDA, strain LUPP2376 was stored in 30% glycerol at −80 °C until use. Previous work noted that strain LUPP2376 was highly pathogenic towards oilseed rape (*Br. napus*), cv. Flash [34], and therefore initial bioactivity trials utilised this susceptible cultivar of *Brassica*. Strain LUPP2376 was defrosted at room temperature and plated onto PDA. Petri plates containing the pathogen were subsequently incubated for two weeks at 15–20 °C with a 16/8 h (light/dark) photoperiod. A spore suspension of the pathogen was then prepared as described for the candidate endophytes, *Be. bassiana* and *G. pannorum*, at a concentration of 10^7^ spores per ml. The viability of *L. maculans* spores was assessed by spraying aliquots of the prepared spore suspension onto fresh PDA and observing the developing colonies after 5 days of incubation at 18 °C in the dark.

Seeds of oilseed rape were surface-disinfected and inoculated with the two fungal endophytes as described earlier. Control seeds were only treated with a sterile aqueous Tween-20^®^ solution. Seeds from all treatment groups were then placed on sterile filter paper to dry, and later transferred to sterile plastic plant containers (product number 2105646, Alto Ltd., Auckland, New Zealand) containing autoclaved vermiculite. Subsequently, seedlings were planted in seedling trays containing autoclaved potting mix within Saxon mini greenhouses (Bunnings Group, Hawthorn East, Australia). The mini greenhouses were closed with a lid, sealed with plastic tape to keep humidity in the tray elevated and placed in a controlled environment (A1000, Conviron Asia Pacific Pty Ltd., Grovedale, Australia) at 18 °C with a 16/8 h (light/dark) photoperiod. At the cotyledon leaf stage, one cotyledon leaf per seedling was punctured with a sterile needle and 15 µL of the *L. maculans* spore suspension was placed on the wound site using a pipette. Plants were incubated, as described earlier, for two weeks to allow disease symptoms to appear and subsequently assessed using a 0–6 scale as described by Hammoudi et al. [35]: 0 = no symptoms; 1 = lesions on the infection site < 1.5 mm; 2 = lesions on the infection site 1.5–3.5 mm; 3 = lesions on the infection site > 3.0 mm; 4 = grey-to-green tissue collapse 3.1–5.0 mm; 5 = grey-to-green tissue collapse > 5.0 mm (≤10 pycnidia); 6 = grey-to-green tissue collapse > 5.0 mm (>10 pycnidia). The mean score from 10 infected seedlings from each tray was used in the analysis. Newly emerging leaves were carefully removed during the experimental period and the experiment was repeated once. There were three treatments (seed treated with two fungal endophytes, and an aqueous Tween-20^®^ solution acting as a pathogen-only control), with 10 seedlings per treatment, arranged in a randomised complete block design.

Statistical analyses were performed using the software package R [32]. A cumulative link mixed model (CLMM) from the “ordinal” R package was used to model the lesion severity scores using an equidistant threshold [36]. The two experiments (1 + 2) were modelled separately. The treatment group was used as a fixed effect. Random intercepts were used for each block. Analysis of Deviance was used to assess the significance of the fixed effects. The R package “emmeans” was used to generate predicted means, standard errors and 95% confidence limits for lesion scores [33]. Multiple comparison p-value adjustment was performed using Tukey’s method.

## 3. Results

### 3.1. Fungal Endophytes Isolated from Wild Brassica

**Figure 1:**
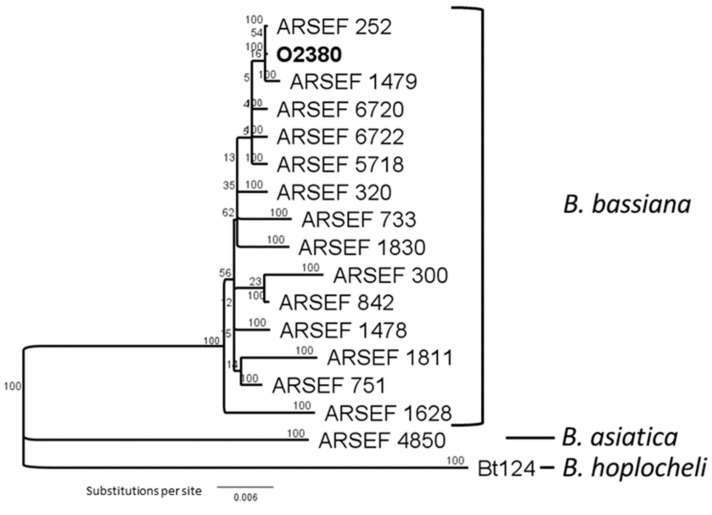
A phylogenetic tree comparing *Be. bassiana* strain O2380 isolated from *Br. rapa* to selected reference isolates using elongation factor sequences, including strains sequenced by Rehner & Buckley [31].

The fungal isolates from *Br. rapa* accessions O2380 and O2377 were identified as *Be. bassiana* and *Pseudogymnoascus pannorum* (syn. *Geomyces pannorum*), respectively. The isolate from accession O2380 displayed slow-growing white colonies on PDA at 22 °C. After 3–4 days incubation, the culture produced single-celled, near-spherical, hyaline conidia formed on a zig-zag conidiophore, or rachis, characteristic of *Beauveria* spp. A phylogenetic tree was produced comparing the *Beauveria* strain isolated from *Br. Rapa* accession O2380 to reference isolates, including strains sequenced by Rehner & Buckley [31]. Several strains of *Be. Bassiana* (*sensu stricto*) were similar to that from accession O2380 (Figure 1), based on partial sequences of the elongation factor gene, confirming its identity.

The fungal isolate from accession O2377 produced single-celled, hyaline, wedge-shaped conidia with a flat base on short conidiophores. These characteristics resembled those of *P. pannorum* and were distinguishable from *P. destructans*, a pathogen of bats that produces sickle-shaped conidia and is unable to grow above 22 °C [37,38,39]. The isolate from accession O2377 grew at temperatures above 22 °C but failed to grow above 37 °C. Subsequent PCR amplification of the ITS rDNA gene sequences confirmed its identity as *P. pannorum*.

### 3.2. Development of Novel Plant–Endophyte Associations

**Figure 2:**
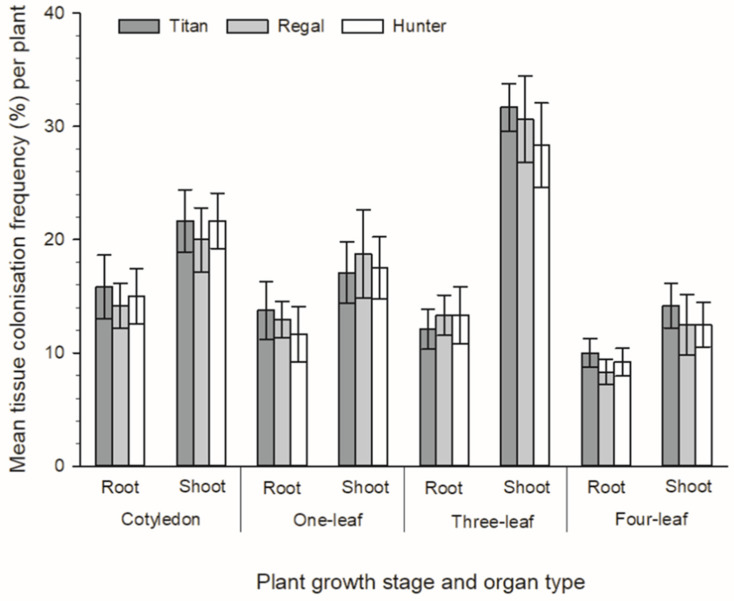
Tissue colonisation frequency (TCF%) of *Pseudogymnoascus pannorum* O2377 within shoot and root tissues of three forage *Brassica* cultivars (rape, cv. Titan, kale, cv. Regal and a leafy turnip, cv. Hunter) at four plant growth stages (cotyledon, one-leaf, two-leaf and four-leaf).

**Figure 3:**
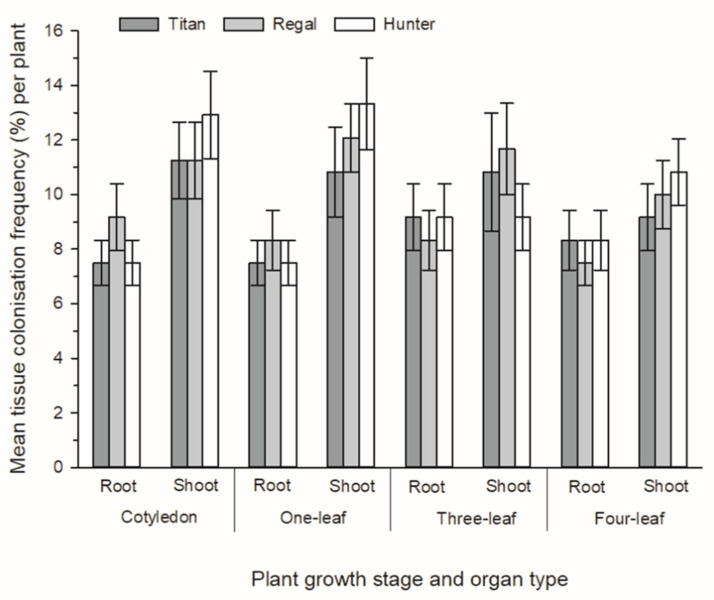
Tissue colonisation frequency (TCF%) of *Beauveria bassiana* O2380 within shoot and root tissues of three forage *Brassica* cultivars (rape, cv. Titan, kale, cv. Regal and a leafy turnip, cv. Hunter) at four plant growth stages (cotyledon, one-leaf, two-leaf and four-leaf).

Both *Be. bassiana* and *P. pannorum* strains were isolated from all plants, and all forage *Brassica* cultivars (rape, cv. Titan, kale, cv. Regal and a leafy turnip, cv. Hunter) that were intentionally inoculated with these endophytic fungi. Additionally, these fungi were isolated from all four plant growth stages (cotyledon, one-leaf, two-leaf and four-leaf) from all plants that were assessed. No plants exhibited any obvious disease symptoms related to colonisation by these two fungi. Overall, there was a greater abundance of *P. pannorum* isolated from the tissue pieces that were sampled from these artificially inoculated plants compared to *Be. bassiana*. When all cultivars were pooled from all plant growth stages, there was a significantly greater colonisation frequency recovered from shoot samples compared to those from the root of both *P. pannorum* and *Be. bassiana* (*X*^2^_1_ = 34.62, *p* < 0.001 and *X*^2^_1_ = 6.82, *p* = 0.009, respectively) (Figure 2 and Figure 3). For *P. pannorum*, this was especially evident at the three-leaf stage, where there was more than twice the number of tissue pieces from the shoot colonised in comparison to those from the root of *Brassica* (*p* = 0.015) and this was consistent across all three cultivars of *Brassica* (Figure 2). There were no effects of cultivar on the colonisation frequency of either *P. pannorum* or *Be. bassiana* (Figure 2 and Figure 3). All uninoculated (control) plants remained free of both *P. pannorum* and *Be. bassiana* (data not shown).

### 3.3. Bioactivity of Fungal Endophytes towards Leptosphaeria Maculans

**Figure 4:**
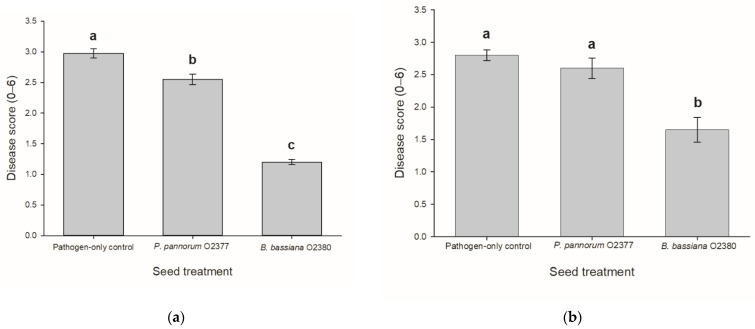
Mean disease score of oilseed rape (*Br. napus*), cv. Flash leaves after wounding and inoculation by *Leptosphaeria maculans* following treatment of seeds by *Pseudogymnoascus pannorum* O2377, *Beauveria bassiana* O2380 or an aqueous Tween-20^®^ solution (pathogen-only control) (±SE). Results are presented from two replicate experiments, Experiment 1 (**a**) and Experiment 2 (**b**). Bars followed by the same letter are not significantly different (*p* < 0.05). Disease scores were assessed using a 0–6 scale whereby 0 = no symptoms on wound site; 1 = lesions on the wound site < 1.5 mm; 2 = lesions on the wound site 1.5–3.5 mm; 3 = lesions on the wound site > 3.0 mm; 4 = grey-to-green tissue collapse 3.1–5.0 mm; 5 = grey-to-green tissue collapse > 5.0 mm (≤10 pycnidia); 6 = grey-to-green tissue collapse > 5.0 mm (>10 pycnidia).

In experiment 1, both *P. pannorum* and *Be. bassiana* significantly (*p* < 0.001, z = −2.63 and *p* < 0.001, z = −7.39, respectively) suppressed the amount of disease caused by *L. maculans* on leaves of oilseed rape (*Br. napus*), cv. Flash after wounding, compared to the pathogen-only control (Figure 4). In experiment 2, only *Be. bassiana* significantly (*p* < 0.001, z = −5.04) suppressed the amount of disease caused by *L. maculans* on leaves of oilseed rape (*Br. napus*) compared to the pathogen-only control (Figure 4).

## 4. Discussion

Our previous research identified that *Methylobacterium* was the dominant cultural bacterial genus inhabiting wild *Brassica* plants [24]. Within the same study, two fungal isolates were also recovered from accessions of wild *Brassica,* but this was not reported earlier. Utilising DNA-based and traditional mycology techniques, these fungal isolates were identified as *Be. bassiana* and *P. pannorum*. Both fungi were exceptionally rare amongst the wild *Brassica* accessions surveyed, only being recovered from single, separate accessions of *Br. rapa*, both originating from California, USA. Both fungi were isolated from multiple root and shoot samples dissected from several symptomless plants generated from surface disinfected seed.

Further study showed that both these fungi could colonise multiple cultivars of forage *Brassica* at high infection frequencies after artificial inoculation of *Brassica* seeds. Additionally, there were no effects of cultivar on the colonisation frequency by these fungal isolates, perhaps indicating a lack of host-specificity within the *Brassica* genus. Both fungi were also recovered from all four of the plant growth stages sampled (cotyledon, one-leaf, two-leaf and four-leaf), from all plants that were assessed, indicating that these fungi formed a self-sustaining and stable association with their plant hosts for many weeks post-inoculation. None of the plants colonised by these fungi exhibited any obvious disease symptoms, indicating the formation of novel mutualistic associations.

This is not the first time that these fungal species have been reported as being closely associated with vascular plants and their seeds. *P. pannorum* (syn. *G. pannorum*) associates with multiple plant species, exhibiting cellulolytic and keratinolytic abilities.

*Pseudogymnoascus* spp. have a global distribution, with most species being saprophytic exhibiting psychrophilic or psychrotolerant capabilities with *P. pannorum* present within temperate soils and permafrost within the Arctic and Antarctic [40,41]. The fungus has been identified from the rhizosphere of peat bog plants, the roots of *Erica arborea* and is described as an endophyte of *Colobanthus quitensis*, *Rhododendron* and *Vaccinium* [42,43,44,45]. Relatives of the fungus, such as species of *Oidiodendron*, are well-known ericoid mycorrhiza fungi that can improve nutrient uptake [44]. Vohník et al. [42] described *P. pannorum* as a putative ericoid mycorrhizal fungus due to its ability to produce intracellular coils in the rhizodermal cells of *Vaccinium* microcuttings. However, no obvious signs of improved fitness were observed for the host. The *P. pannorum* isolate from our study colonised shoot samples to a higher degree than the root. We did not investigate the formation of specialised structures within the original wild host accession or within artificial associations developed with the *Brassica* cultivars, but this will be incorporated in any future work.

Although *Be. bassiana* (family, Cordycipitaceae), is well-known for its association with insects and insect habitats, the species has been described as an endophyte of many dicot and monocot plant species [46]. Economically important crops endophytically colonised by the fungus include banana [47], broad bean [48], cauliflower [49], cocoa [50], cotton [51], grape [52], maize [53,54], pine [55], sorghum [56], sugarcane [57], tomato [58] and wheat [59]. Across this diverse host range, *Be. bassiana* has been observed colonising both vegetative (i.e., leaves, shoots and roots) and reproductive (i.e., seed) plant organs [51,60] providing further evidence for its mutualistic relationship with plants. Further microscopy study is required in order to determine whether this strain of *Be. bassiana* is endophytic within *Brassica* spp., as some fungal strains possess a saprophytic and epiphytic lifestyle as opposed to an endophytic one [61].

*Beauveria* spp. are entomopathogenic fungi. *Be. bassiana* is the most widely known member of the genus and is responsible for white muscardine disease, which affects various arthropod species [62]. For this reason, *Be. bassiana* has previously been developed as a biopesticide, with many products used in the biological control of a wide range of invertebrate pests including aphids, beetles, caterpillars, termites, thrips and whitefly [62,63,64]. The insect-killing ability of *Be. bassiana* has been known for around 200 years [65], however additional behaviours exhibited by the fungus have only been recently identified. These behaviours include the mutualistic symbiosis formed with many plant species. Traits conferred to plant hosts by *Be. Bassiana* are analogous to more commonly recognised plant symbionts, and include antifungal activity and plant promotional traits via mechanisms such as phosphate solubilization and siderophore production [66]. However, not all plant associations result in beneficial effects against invertebrates or plant pathogens [63]. The novel association formed between *Brassica* plants and *Be. bassiana* significantly inhibited phoma stem canker, a devastating disease of *Brassica* crops worldwide, caused by the fungal pathogen *L. maculans*. The novel association formed with *P. pannorum* significantly suppressed the amount of disease caused by *L. maculans* in one out of two experiments.

The teleomorphs of many *Beauveria* species are species of *Cordyceps*, with the teleomorph of *Be. bassiana* being *Cordyceps bassiana,* which has only been discovered in eastern Asia so far [67]. *Be. bassiana* and *Be. brongniartii* are the only species from this genus that have been shown to be endophytic [68]. However, Rehner and Buckley [31] report that *Be. bassiana* can be divided into two unrelated and morphologically indistinguishable clades formally described as *Be. bassiana* and *Be. pseudobassiana*. *Be. bassiana sensu stricto* is generally recognised as a globally distributed, genetically diverse species or species complex [69,70].

To our knowledge, this is the first study published on *Be. bassiana* providing protection against *L. maculans*, although strains of the entomopathogen have been reported to exhibit antifungal activity towards other phytopathogenic fungi. Pus [71] assessed three strains of *Be. bassiana* for their ability to control several pests and diseases of cabbage (*Brassica oleracea* var. *capitata*). Two strains of *Be. bassiana* significantly reduced the lifespan of green peach aphid (*Myzus persicae*) and disease caused by *Sclerotinia sclerotiorum,* but none inhibited infection caused by *L. maculans* [71]. Strains of *Be. Bassiana* have also provided potato with protection from *Rhizoctonia solani* [72] and tomato with protection from *Fusarium oxysporum* f. sp. *lycopersici* [73], *Rhizoctonia solani* and *Pythium myriotylum* [51]. Further antifungal activity has been observed in vitro towards a wider number of plant pathogens (see [51] for an extensive list). Mechanisms attributed to this bioactivity include antibiosis via multiple secondary metabolite compounds, competition, direct parasitism, and induced resistance [51,74,75,76]. We are yet to identify the mechanism/s attributed to the *Be. bassiana* strain investigated within our study.

## 5. Conclusions

Rapeseed is now the most widely cultivated crop in the family Brassicaceae and the third-most abundant oil crop worldwide [77]. Foliar fungicide applications to control *L. maculans* have been proven to be of limited value, as resistance is increasing in certain populations [78,79]. Furthermore, the timing of fungicide application is crucial, as minimal disease control is achieved once the pathogen has reached the plant stem. *Be. bassiana* as an endophyte offers an alternative, effective delivery mechanism for this biological control agent of invertebrate pests and plant pathogenic bacteria and fungi [47]. A biocontrol agent with dual protection, from herbivory and disease, increases the marketability of a product based on the fungus [51,80,81]. Additionally, *Be. bassiana*, like several other species within the order Hypocreales has a wide host range and is amenable to mass production [82]. With pesticides being withdrawn from many markets, driven by consumer demands for pesticide-free produce that is more sustainable while limiting damage to the environment, alternative pest and disease management options need to be addressed. Although biological control is not a new strategy, new techniques coupled with greater knowledge around the interactions between microbes and their hosts makes endophytic fungi a highly conceivable option to manage pests and diseases of important crops.

## Data Availability

All data are included in the present study.
